# A deterministic mathematical model for bidirectional excluded flow with Langmuir kinetics

**DOI:** 10.1371/journal.pone.0182178

**Published:** 2017-08-23

**Authors:** Yoram Zarai, Michael Margaliot, Tamir Tuller

**Affiliations:** 1 Dept. of Biomedical Engineering, Tel-Aviv University, Tel-Aviv 69978, Israel; 2 School of Electrical Engineering and the Sagol School of Neuroscience, Tel-Aviv University, Tel-Aviv 69978, Israel; 3 Dept. of Biomedical Engineering and the Sagol School of Neuroscience, Tel-Aviv University, Tel-Aviv 69978, Israel; Ben-Gurion University, ISRAEL

## Abstract

In many important cellular processes, including mRNA translation, gene transcription, phosphotransfer, and intracellular transport, biological “particles” move along some kind of “tracks”. The motion of these particles can be modeled as a one-dimensional movement along an ordered sequence of sites. The biological particles (e.g., ribosomes or RNAPs) have volume and cannot surpass one another. In some cases, there is a preferred direction of movement along the track, but in general the movement may be bidirectional, and furthermore the particles may attach or detach from various regions along the tracks. We derive a new deterministic mathematical model for such transport phenomena that may be interpreted as a dynamic mean-field approximation of an important model from mechanical statistics called the asymmetric simple exclusion process (ASEP) with Langmuir kinetics. Using tools from the theory of monotone dynamical systems and contraction theory we show that the model admits a unique steady-state, and that every solution converges to this steady-state. Furthermore, we show that the model entrains (or phase locks) to periodic excitations in any of its forward, backward, attachment, or detachment rates. We demonstrate an application of this phenomenological transport model for analyzing ribosome drop off in mRNA translation.

## Introduction

Movement is essential for cell function. Cargoes like organelles and vesicles must be carried between different locations in the cells. The information encoded in DNA and mRNA molecules must be decoded by “biological machines” (RNA polymerases and ribosomes) that move along these molecules sequentially.

**Fig 1:**
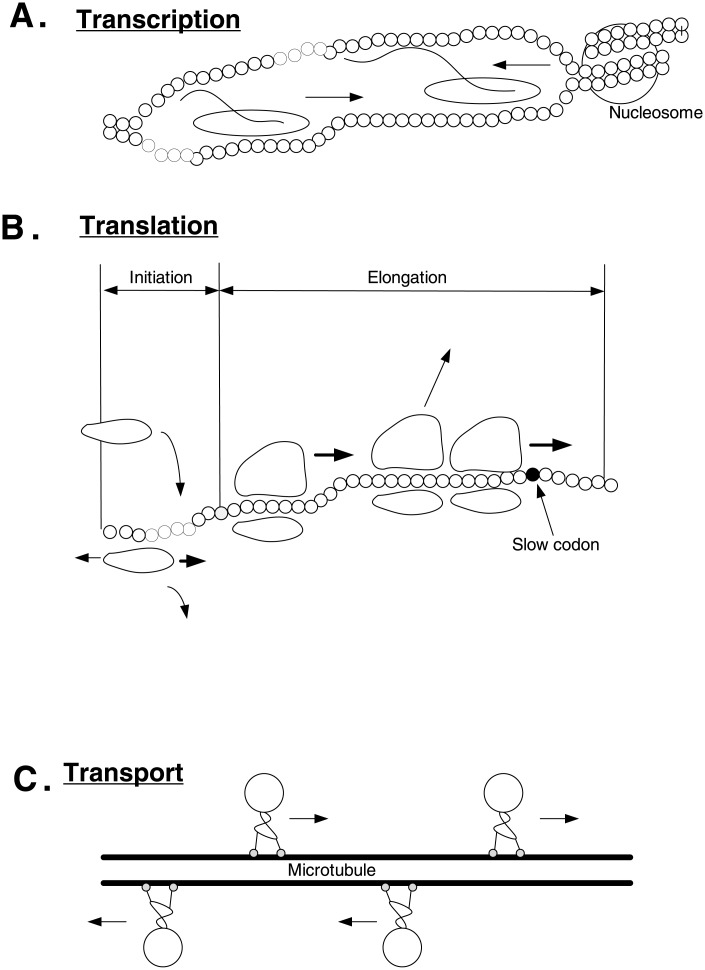
Biological processes that can be studied using the model derived in this paper. A—transcription of a DNA gene into messenger RNA (mRNA) by RNA polymerase. B—mRNA translation by macromolecules called ribosomes. C—intracellular transport by motor proteins.

Many of these important biological transport processes are modeled as the movement of particles along an ordered chain of sites. In the context of intracellular transport, the particles are motor proteins and the chain models actin filaments or microtubules. In transcription, the particles are RNAPs moving along the DNA molecule, and in translation the particles are ribosomes moving along the mRNA molecule (see Fig 1).

The movement in such processes may be unidirectional, as in mRNA translation elongation, or bidirectional, as in transcription or translation initiation. Indeed, the normal forward flow of the RNAP may be interrupted, due to transcription errors and various obstacles such as nucleosomes, in which case the RNAP tracks back a few nucleotides and then resumes its normal forward flow [1–4]. Translation initiation in eukaryotes usually includes diffusion from the 5’end of the transcript towards the start codon [5]. This diffusion process is believed to be bidirectional, but with a preference to the 5’→3’ direction. The movement of motor proteins like kinesin and dynein along microtubules is typically unidirectional, but can be bidirectional as well [5].

To increase efficiency, many particles may move simultaneously along the same track thus pipelining the production process. For example, to increase translation yield, a number of ribosomes may act simultaneously as polymerases on the same mRNA molecule [6, 7].

The moving biological particles have volume and usually cannot overtake a particle in front of them. This means that a slowly moving particle may lead to the formation of a traffic jam behind it. For example, Leduc et al. [8] have studied Kip3, a yeast kinesin-8 family motor, and demonstrated that motor protein traffic jams can exist, given the right conditions. Other studies have suggested that traffic jams of RNAP [ribosomes] may evolve during transcription [translation] [7, 9, 10].

In some of these biological transport processes the biological machines may either attach or detach at various sites along the tracks. For example, ribosomes may detach from the mRNA molecule before reaching the stop codon due to traffic jams and ribosome-ribosome interactions or due to depletion in the concentration of tRNAs [11–13]. Also, it is known that kinesin-family motor proteins are more susceptible to dissociation when their path is blocked [14, 15]. Defects in these transport processes may lead to severe diseases or may even be lethal. For example, [16] lists the implications of malfunctions of protein motors in disease and developmental defects.

Developing a better understanding of these dynamical biological processes by combining mathematical modeling and biological experiments will have far reaching implications to basic science in fields such as molecular evolution and functional genomics, as well as applications in synthetic biology, biotechnology, human health, and more. Mathematical or computational modeling is especially important in developing approaches for manipulating and controlling these processes, e.g. in order to optimize various goals in biotechnology.

A standard model for such transport processes is the *asymmetric simple exclusion process* (ASEP) [17, 18]. This is a stochastic model describing particles that hop along an ordered lattice of sites. Each site can be either empty or occupied by a single particle, and a particle can only hop to an empty site. This *simple exclusion principle* represents the fact that the particles have volume and cannot overtake one another. Simple exclusion generates an indirect coupling between the particles. In particular, traffic jams may develop behind a slow-moving particle.

The motion is bidirectional i.e. a particle may hop to any of the two neighboring sites (but only if they are free) and asymmetric, that is, there may be a preferred direction of motion. Typically, a particle can attach to the lattice in one of its ends and detach from the other end. When particles can also attach or detach at internal sites along the lattice, the model is referred to as ASEP with *Langmuir kinetics*. In the special case where the hops are unidirectional, ASEP is sometimes referred to as the *totally asymmetric simple exclusion process* (TASEP). A TASEP-like system with Langmuir kinetics has been used to model limit order markets in [19], and is often used in modeling molecular motor traffic [20–24]. For TASEP with open boundary conditions (i.e. when the two sides of the lattice are connected to two particle reservoirs, as assumed in this paper) and with Langmuir kinetics, no exact solutions are known. The phase diagram and the shock formation of the homogeneous TASEP (i.e. where all the internal hopping rates are assumed to be equal) with Langmuir kinetics in the thermodynamical limit, that is as the number of lattice sites goes to infinity, was analyzed in [20, 21, 25] using a mean-field approximation. It was shown that the phase diagram is much richer than that of TASEP because phase coexistence becomes possible due to the Langmuir kinetics. Homogeneous TASEP with periodic boundary conditions (i.e. when the lattice forms a ring) and with Langmuir kinetics was analyzed in [24–26].

More generally, ASEP has become a fundamental model in non-equilibrium statistical mechanics, and has been applied to model numerous natural and artificial processes including traffic and pedestrian flow, the movement of ants, evacuation dynamics, and more [27].

In this paper, we introduce a deterministic mathematical model that may be interpreted as a dynamic *mean-field approximation of ASEP with Langmuir kinetics* (MFALK) [25]. We analyze the MFALK using tools from systems and control theory. In particular, we apply some recent developments in contraction theory to prove that the model is globally asymptotically stable, and that it entrains to periodic excitations in the transition/attachment/detachment rates. In other words, if these rates change periodically in time with some common period *T* then all the state-variables in the MFALK converge to a periodic solution with period *T*. This is important because many biological processes are excited by periodic signals (e.g. the 24h solar day or the periodic cell-division process), and proper functioning requires phase-locking or entrainment to these excitations.

Our work is motivated by the analysis of a model for mRNA translation called the *ribosome flow model (RFM)* [28]. This is a mean-field approximation of the *unidirectional* TASEP *without* Langmuir kinetics (see, e.g., section 4.9.7 in [27] and p. R345 in [29]). Recently, the RFM has been studied extensively using tools from systems and control theory [30–40]. The analysis is motivated by implications to many important biological questions. For example, the sensitivity of the protein production rate to the initiation and elongation rates along the mRNA molecule [36], maximization of protein production rate [35], the effect of ribosome recycling [33, 37], and the consequences of competition for ribosomes on large-scale simultaneous mRNA translation in the cell [41] (see also [42, 43] for some related models).

The MFALK presented here is much more general than the RFM, and can thus be used to model and analyze many transport phenomena, including all the biological processes mentioned above, that cannot be captured using the RFM. We demonstrate this by using the MFALK to model and analyze mRNA translation with *ribosome drop off*—an important feature that cannot be modeled using the RFM.

Ribosome drop off is a fundamental phenomena that has received considerable attention (see, e.g., [12, 13, 44–51]). In many cases, ribosome drop off is deleterious to the cell since translation is the most energetically consuming process in the cell and, furthermore, drop off yields truncated, non-functional proteins. Thus, transcripts undergo selection to minimize drop off or its energetic cost [12, 48, 49, 51–53]. There are various hypotheses on the biological advantages of ribosome drop off. For example, Zaher and Green [54] have suggested that ribosome drop off is related to proof reading. One may perhaps expect that another advantage is that drop off from a jammed site may increase the total flow by reducing congestion. Our analysis of the MFALK shows that this is not true. Drop off has a substantial effect on the flow, yet it always leads to a reduction in the steady-state protein production rate.

The remainder of this paper is organized as follows. The next section describes the new mathematical model. Section 2 presents our main analysis results. Section 3 describes the application of the MFALK to model mRNA translation with ribosome drop off. The final section concludes and describes possible directions for further research. To streamline the presentation, all the proofs are placed in the S1 File.

## 1 The model

The MFALK is a set of *n* first-order nonlinear differential equations, where *n* denotes the number of compartments or sites along the track. Each site is associated with a state variable *x*_*i*_(*t*) ∈ [0, 1] describing the normalized “level of occupancy” (or density) at site *i* at time *t*, with *x*_*i*_(*t*) = 0 [*x*_*i*_(*t*) = 1] representing that site *i* is completely free [full] at time *t*. Since *x*_*i*_(*t*) ∈ [0, 1] for all *t*, it may also be interpreted as the probability that site *i* is occupied at time *t*.

The MFALK contains four sets of non-negative parameters:

- λ_*i*_, *i* = 0, …, *n*, controls the forward transition rate from site *i* to site *i* + 1,
- *γ*_*i*_, *i* = 0, …, *n*, controls the backward transition rate from site *i* + 1 to site *i*,
- *β*_*i*_, *i* = 1, …, *n*, controls the attachment rate to site *i*,
- *α*_*i*_, *i* = 1, …, *n*, controls the detachment rate from site *i*,

where we arbitrarily refer to left-to-right flow along the chain as forward flow, and to flow in the other direction as backward flow. Each parameter λ_*i*_, *γ*_*i*_, *α*_*i*_ and *β*_*i*_ has units of 1/time.

The dynamical equations describing the MFALK are:

$$\begin{array}{ccc} {\dot{x}}_{1} & = & \lambda_{0}(1-x_{1})+\gamma_{1}x_{2}(1-x_{1})+\beta_{1}(1-x_{1})-\lambda_{1}x_{1}(1-x_{2})-\gamma_{0}x_{1}-\alpha_{1}x_{1},\\ {\dot{x}}_{2} & = & \lambda_{1}x_{1}\left(1-x_{2}\right)+\gamma_{2}x_{3}\left(1-x_{2}\right)+\beta_{2}\left(1-x_{2}\right)-\lambda_{2}x_{2}\left(1-x_{3}\right)-\gamma_{1}x_{2}\left(1-x_{1}\right)-\alpha_{2}x_{2},\\ \vdots & & \\ {\dot{x}}_{n-1} & = & \lambda_{n-2}x_{n-2}(1-x_{n-1})+\gamma_{n-1}x_{n}(1-x_{n-1})+\beta_{n-1}(1-x_{n-1})-\lambda_{n-1}x_{n-1}(1-x_{n})\\ & - & \gamma_{n-2}x_{n-1}(1-x_{n-2})-\alpha_{n-1}x_{n-1},\\ {\dot{x}}_{n} & = & \lambda_{n-1}x_{n-1}(1-x_{n})+\gamma_{n}(1-x_{n})+\beta_{n}(1-x_{n})-\lambda_{n}x_{n}-\gamma_{n-1}x_{n}(1-x_{n-1})-\alpha_{n}x_{n}. \end{array}$$ (1)

To explain these equations, consider for example the equation for the change in the occupancy in site 2, namely,

$$\begin{array}{c} {\dot{x}}_{2}=\lambda_{1}x_{1}\left(1-x_{2}\right)+\gamma_{2}x_{3}\left(1-x_{2}\right)+\beta_{2}\left(1-x_{2}\right)-\lambda_{2}x_{2}\left(1-x_{3}\right)-\gamma_{1}x_{2}\left(1-x_{1}\right)-\alpha_{2}x_{2}. \end{array}$$

**Fig 2:**
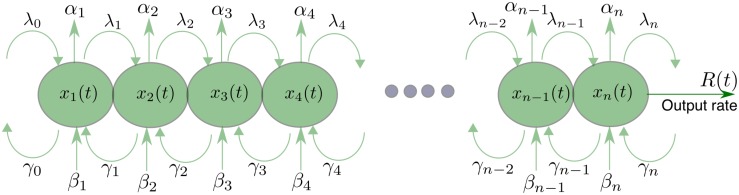
Topology of the MFALK. The state variable *x*_*i*_(*t*) ∈ [0, 1] describes the density of site *i* at time *t*. The parameter λ_*i*_ [*γ*_*i*_] controls the transition rate from site *i* [*i* + 1] to site *i* + 1 [*i*]. The parameter *α*_*i*_ [*β*_*i*_] controls the detachment [attachment] rate from [to] site *i*. *R*(*t*) denotes the output rate at time *t*.

The term λ_1_*x*_1_(1 − *x*_2_) represents the flow from site 1 to site 2. This increases with the occupancy in site 1, and decreases with the occupancy in site 2. In particular, this term becomes zero when *x*_2_ = 1, i.e. when site 2 is completely full. This is a “soft” version of the hard exclusion principle in ASEP: the effective entry rate into a site decreases as it becomes fuller. Note that the constant λ_1_ ≥ 0 describes the maximal possible transition rate from site 1 to site 2. Similarly, the term λ_2_*x*_2_(1 − *x*_3_) represents the flow from site 2 to site 3. The term *γ*_2_*x*_3_(1 − *x*_2_) [*γ*_1_*x*_2_(1 − *x*_1_)] represents the backward flow from site 3 to site 2 [site 2 to site 1]. Note that these terms also model soft exclusion. The term *β*_2_(1 − *x*_2_) represents attachment of particles from the environment to site 2, whereas *α*_2_*x*_2_ represents detachment of particles from site 2 to the environment (see Fig 2). The other equations can be explained similarly.

The MFALK is a *compartmental model* [55, 56], as every state-variable describes the occupancy in a compartment (e.g., a site along the mRNA, gene, microtubule), and the dynamical equations describe the flow between these compartments and also with the environment. Compartmental models play an important role in pharmacokinetics, enzyme kinetics, basic nutritional processes, cellular growth, and pathological processes, such as tumourigenesis and atherosclerosis (see, e.g., [55, 57] and the references therein). More specifically, the MFALK is a nonlinear tridiagonal compartmental model, as every ${\dot{x}}_{i}$ directly depends on *x*_*i*−1_, *x*_*i*_, and *x*_*i*+1_ only.

Note also that

$$\begin{array}{ll} \sum_{i=1}^{n}{\dot{x}}_{i} & =\lambda_{0}(1-x_{1})-\gamma_{0}x_{1}+\beta_{1}(1-x_{1})-\alpha_{1}x_{1}\\ & +\gamma_{n}(1-x_{n})-\lambda_{n}x_{n}+\beta_{n}(1-x_{n})-\alpha_{n}x_{n}\\ & +\sum_{i=2}^{n-1}\left(\beta_{i}(1-x_{i})-\alpha_{i}x_{i}\right). \end{array}$$ (2)

The term on the right-hand side of the first [second] line here represents the change in *x*_0_ [*x*_*n*_] due to the flow between the environment and site 1 [site *n*], whereas the term on the third line represents the flow between internal sites and the environment.

The *output rate* from site *n* at time *t* is the total flow from this site to the environment:

$$R(t)≔\left(\lambda_{n}+\alpha_{n}\right)x_{n}\left(t\right)-\left(\gamma_{n}+\beta_{n}\right)\left(1-x_{n}\left(t\right)\right).$$ (3)

Note that *R*(*t*) may be positive, zero, or negative.

In the particular case where *α*_*i*_ = *β*_*i*_ = *γ*_*i*_ = 0 for all *i* the MFALK becomes the RFM, i.e. a dynamic mean-field approximation of the unidirectional TASEP with open boundary conditions and without Langmuir kinetics.

Let *x*(*t*, *a*) denote the solution of Eq (1) at time *t* ≥ 0 for the initial condition *x*(0) = *a*. Since the state-variables correspond to normalized occupancy levels, we always assume that *a* belongs to the closed *n*-dimensional unit cube:

$$\begin{array}{c} C^{n}≔\left\{x\in R^{n}:x_{i}\in \left[0,1\right],i=1,\ldots ,n\right\}. \end{array}$$

Let int(*C*^*n*^) denote the interior of *C*^*n*^, and let ∂*C*^*n*^ denote the boundary of *C*^*n*^. The next section analyzes the MFALK defined in Eq (1).

## 2 Main results

For notational convenience, let *α*_0_ ≔ 0, *γ*_0_ ≔ 0, *α*_*n*+1_ ≔ 0, and *β*_*n*+1_ ≔ 0. Recall that all the proofs are placed in S1 File.

### 2.1 Invariance and persistence

It is straightforward to show that *C*^*n*^ is an invariant set for the dynamics of the MFALK, that is, if *a* ∈ *C*^*n*^ then *x*(*t*, *a*) ∈ *C*^*n*^ for all *t* ≥ 0. The following result shows that a stronger property holds.

**Proposition 1** *Suppose that at least one of the following two conditions holds*:

$$\begin{array}{c} \lambda_{i}+\beta_{i+1}>0,\;for\;all\;i\in \left\{0,\ldots ,n\right\}, \end{array}$$ (4)

*or*

$$\begin{array}{c} \gamma_{i}+\alpha_{i+1}>0,\;for\;all\;i\in \left\{0,\ldots ,n\right\}. \end{array}$$ (5)

*Then for any* *τ* > 0 *there exists* *d* = *d*(*τ*) ∈ (0, 1/2] *such that*

$$\begin{array}{c} d\leq x_{i}\left(t+\tau ,a\right)\leq 1-d, \end{array}$$ (6)

*for all* *a* ∈ *C*^*n*^, *all* *i* ∈ {1, …, *n*}, *and all* *t* ≥ 0.

This means in particular that trajectories that emanate from the boundary of *C*^*n*^ “immediately” enter *C*^*n*^, and also that every occupancy is “immediately” uniformly separated from zero and from one. This result is useful because as we will see below on the boundary of *C*^*n*^ the MFALK loses some important properties. For example, the Jacobian matrix of the dynamics Eq (1) is irreducible on int(*C*^*n*^), but becomes reducible at some points on the boundary of *C*^*n*^.

### 2.2 Contraction

Differential analysis and in particular contraction theory proved to be a powerful tool for analyzing nonlinear dynamical systems. In a contractive system, trajectories that emanate from different initial conditions contract to each other at an exponential rate [58–60]. Let ${\left|\cdot \right|}_{1}:R^{n}\to R_{+}$ denote the *L*_1_ norm, i.e. for $z\in R^{n}$, |*z*|_1_ = |*z*_1_| + ⋯ + |*z*_*n*_|.

**Proposition 2** *Let*

$$\begin{array}{c} \eta ≔\max\{-\lambda_{0}-\gamma_{0}-\alpha_{1}-\beta_{1},-\alpha_{2}-\beta_{2},\ldots ,-\alpha_{n-1}-\beta_{n-1},-\lambda_{n}-\gamma_{n}-\alpha_{n}-\beta_{n}\}. \end{array}$$

*Note that* *η* ≤ 0. *For any* *a*, *b* ∈ *C*^*n*^ *and any* *t* ≥ 0,

$$\begin{array}{c} {\left|x\left(t,a\right)-x\left(t,b\right)\right|}_{1}\leq \exp\left(\eta t\right){\left|a-b\right|}_{1}. \end{array}$$ (7)

This means the following. Consider two ribosomal densities *a*, *b* ∈ *C*^*n*^. Define the distance between densities using the *L*_1_ distance: |*a* − *b*|_1_ = |*a*_1_ − *b*_1_| + ⋯ + |*a*_*n*_ − *b*_*n*_|, i.e. the sum of the absolute differences at each site. Consider two time evolutions of the MFALK *x*(*t*, *a*) and *x*(*t*, *b*), i.e. the solution at time *t* when initialized with *x*(0) = *a* and with *x*(0) = *b*. Then the difference between *x*(*t*, *a*) and *x*(*t*, *b*) decreases with time with an exponential rate *η*. Thus, as time progresses the MFALK “quickly forgets” the initial condition and, as we will see below, the density always converges to the same steady-state density.

The value *η* depends on the MFALK parameters and increasing all the sums *α*_*i*_ + *β*_*i*_, *i* = 1, …, *n*, makes the system “more contractive”. Indeed, these parameters have a direct stabilizing effect on the dynamics of site *i*, whereas the other parameters affect the site indirectly via the coupling to the two adjacent sites.

When *η* = 0, Eq (7) only implies that the *L*_1_ distance between trajectories does not increase. This is not strong enough to prove the asymptotic properties described in the subsections below. Indeed, in this case it is possible that the MFALK will *not* be contractive with respect to any fixed norm. Fortunately, a certain generalization of contraction turns out to hold in this case.

Consider the time-varying dynamical system

$$\begin{array}{c} \dot{x}\left(t\right)=f\left(t,x\left(t\right)\right), \end{array}$$ (8)

whose trajectories evolve on a compact and convex set $\Omega \subset R^{n}$. Let *x*(*t*, *t*_0_, *a*) denote the solution of Eq (8) at time *t* for the initial condition *x*(*t*_0_) = *a*. System (8) is said to be *contractive after a small overshoot* (SO) [61] on Ω with respect to (w.r.t.) a norm $|\cdot |:R^{n}\to R_{+}$ if for any *ε* > 0 there exists *ℓ* = *ℓ*(*ε*) > 0 such that

$$\begin{array}{c} |x\left(t,t_{0},a\right)-x\left(t,t_{0},b\right)|\leq \left(1+\varepsilon \right)\exp\left(-\ell t\right)\left|a-b\right|, \end{array}$$

for all *a*, *b* ∈ Ω and all *t* ≥ *t*_0_ ≥ 0. Intuitively speaking, this means contraction with an exponential rate, but with an arbitrarily small overshoot of 1 + *ε*. The next result shows that the MFALK satisfies this generalization of contraction.

**Proposition 3** *Suppose that*

$$\begin{array}{c} \lambda_{i}+\gamma_{i}>0,\;for\;all\;i\in \left\{1,\ldots ,n-1\right\}, \end{array}$$ (9)

*and that at least one of the two conditions* (4) and (5) *holds*. *Then the MFALK is SO on* *C*^*n*^ *w.r.t. the* *L*_1_ *norm*, *that is*, *for any* *ε* > 0 *there exists* *ℓ* = *ℓ*(*ε*) > 0 *such that*

$$\begin{array}{c} {\left|x\left(t,a\right)-x\left(t,b\right)\right|}_{1}\leq \left(1+\varepsilon \right)\exp\left(-\ell t\right){\left|a-b\right|}_{1}, \end{array}$$ (10)

*for all* *a*, *b* ∈ *C*^*n*^ *and all* *t* ≥ 0.

Note that if λ_*i*_ + *γ*_*i*_ = 0 for some *i* ∈ {1, …, *n* − 1}, that is λ_*i*_ = *γ*_*i*_ = 0, then the MFALK decouples into two separate MFALKs: one containing sites 1, …, *i*, and the other containing sites *i*+1, …, *n*. Thus, assuming Eq (9) incurs no loss of generality.

There is an important difference between Propositions 2 and 3. If *η* < 0 then Proposition 2 provides an explicit exponential contraction rate. If *η* = 0 then Proposition 3 can be used to deduce SO, but in this result the contraction rate *ℓ* depends on *ε* and is not given explicitly.

The contraction results above imply that the MFALK satisfies several important and useful asymptotic properties. These are described in the following subsections.

### 2.3 Global asymptotic stability

Since the compact and convex set *C*^*n*^ is an invariant set of the dynamics, it contains a steady-state point *e*. By Proposition 1, *e* ∈ int(*C*^*n*^). Applying Eq (10) with *b* = *e* yields the following result.

**Corollary 1** *Suppose that the conditions in Proposition 3 hold*. *Then the MFALK admits a unique steady-state* *e* ∈ int(*C*^*n*^) *that is globally asymptotically stable*, *i.e.* lim_*t* → ∞_ *x*(*t*, *a*) = *e*, *for all* *a* ∈ *C*^*n*^.

This means that the rates determine a unique density profile along the lattice, and that all trajectories emanating from different initial conditions in *C*^*n*^ asymptotically converge to this density. Thus, any set of rate values λ_*i*_, *γ*_*i*_, *α*_*i*_, and *β*_*i*_ is associated with a unique steady-state density and any solution of the MFALK converges to this density, regardless of the initial density. In addition, perturbations in the occupancy levels along the sites will not change this asymptotic behavior of the dynamics. This also means that various numerical solvers of ODEs will work well for the MFALK (see e.g. [62]).

**Fig 3:**
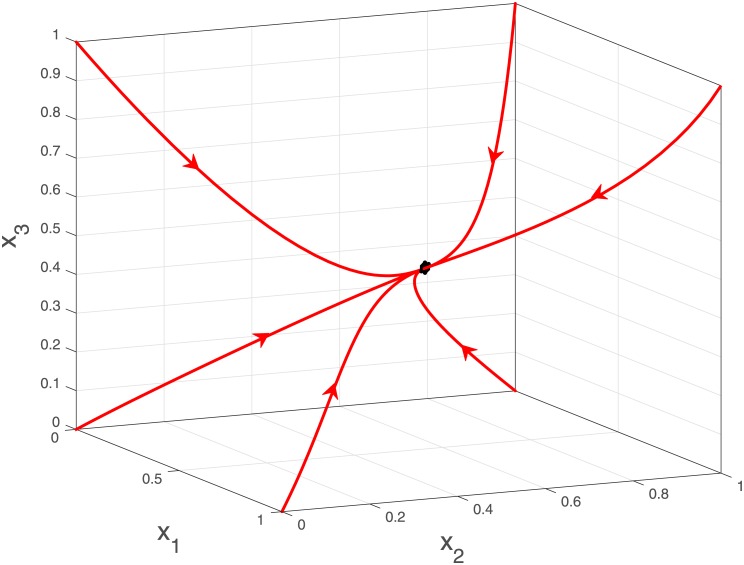
Trajectories of the MFALK in Example 1 for six initial conditions in *C*^3^.

**Example 1** Fig 3 depicts the trajectories of a MFALK with *n* = 3, λ_0_ = 1.0, λ_1_ = 1.2, λ_2_ = 0.8, λ_3_ = 0.9, *γ*_*i*_ = λ_*i*_ − 0.3, *i* = 0, …, 3, *α*_1_ = 0, *α*_2_ = 0.1, *α*_3_ = 0, *β*_1_ = 0, *β*_2_ = 0.2, *β*_3_ = 0, for six initial conditions in *C*^*n*^. It may be seen that all trajectories converge to the same steady-state *e* ∈ int(*C*^3^).

The MFALK Eq (1) can be written as

$$\begin{array}{c} {\dot{x}}_{i}=f_{i-1}\left(x\right)-f_{i}\left(x\right)+g_{i}\left(x_{i}\right),\;i=1,\ldots ,n, \end{array}$$ (11)

where

$$\begin{array}{ccc} f_{0}(x) & ≔ & \lambda_{0}(1-x_{1})-\gamma_{0}x_{1},\\ f_{i}(x) & ≔ & \lambda_{i}x_{i}(1-x_{i+1})-\gamma_{i}x_{i+1}(1-x_{i}),\;i=1,\ldots ,n-1,\\ f_{n}(x) & ≔ & \lambda_{n}x_{n}-\gamma_{n}(1-x_{n}),\\ g_{i}(x_{i}) & ≔ & \beta_{i}(1-x_{i})-\alpha_{i}x_{i},\;i=1,\ldots ,n. \end{array}$$ (12)

At steady-state, i.e. for *x* = *e*, the left-hand side of all the equations in Eq (11) is zero, so

$$\begin{array}{c} f_{i-1}\left(e\right)=f_{i}\left(e\right)-g_{i}\left(e_{i}\right),\;i=1,\ldots ,n. \end{array}$$ (13)

Let $v≔{\left[\begin{array}{c} \alpha_{1},\ldots ,\alpha_{n},\beta_{1},\ldots ,\beta_{n},\gamma_{0},\ldots ,\gamma_{n},\lambda_{0},\ldots ,\lambda_{n} \end{array}\right]}^{\prime }\in {\mathbb{R}}_{+}^{4n+2}$ denote the vector of parameters of the MFALK. It follows from Eq (13) that if we multiply all these parameters by *c* > 0 then *e* will not change, that is, *e*(*cv*) = *e*(*v*). Let

$$\begin{array}{c} R≔\left(\lambda_{n}+\alpha_{n}\right)e_{n}-\left(\gamma_{n}+\beta_{n}\right)\left(1-e_{n}\right), \end{array}$$ (14)

denote the *steady-state output rate*. Then *R*(*cv*) = *cR*(*v*), for all *c* > 0, that is, the steady-state production rate is homogeneous of order one w.r.t. the parameters. By Eq (13),

$$\begin{array}{cc} R & =f_{n}\left(e\right)-g_{n}\left(e_{n}\right)\\ & =f_{i}\left(e\right)+\sum_{j=i+1}^{n-1}g_{j}\left(e_{j}\right),\;i=0,\ldots ,n-1. \end{array}$$ (15)

This yields the following set of recursive equations relating the steady-state occupancy levels and output rate in the MFALK:

$$\begin{array}{cc} e_{n} & =\frac{R+\gamma_{n}+\beta_{n}}{\lambda_{n}+\gamma_{n}+\beta_{n}+\alpha_{n}},\\ e_{i} & =\frac{R+\gamma_{i}e_{i+1}-\sum_{j=i+1}^{n-1}g_{j}(e_{j})}{\lambda_{i}(1-e_{i+1})+\gamma_{i}e_{i+1}},\;i=n-1,\ldots ,1,\\ & \text{and also}\\ e_{1} & =\frac{\lambda_{0}+\beta_{1}-R+\sum_{j=2}^{n-1}g_{j}(e_{j})}{\lambda_{0}+\gamma_{0}+\beta_{1}+\alpha_{1}}. \end{array}$$ (16)

For a given *v*, this is a set of *n* + 1 equations in the *n* + 1 unknowns: *e*_1_, …, *e*_*n*_, *R*.

**Example 2** Consider the MFALK with dimension *n* = 2. Then Eq (16) becomes

$$\begin{array}{cc} e_{2} & =\frac{R+\gamma_{2}+\beta_{2}}{\lambda_{2}+\gamma_{2}+\alpha_{2}+\beta_{2}},\\ e_{1} & =\frac{R+\gamma_{1}e_{2}}{\lambda_{1}(1-e_{2})+\gamma_{1}e_{2}},\\ & \text{and also}\\ e_{1} & =\frac{\lambda_{0}+\beta_{1}-R}{\lambda_{0}+\gamma_{0}+\beta_{1}+\alpha_{1}}. \end{array}$$ (17)

This yields the polynomial equation *a*_2_*R*^2^ + *a*_1_*R* + *a*_0_ = 0, where

$$\begin{array}{l} a_{2}≔\lambda_{1}-\gamma_{1},\\ a_{1}≔(\lambda_{1}-\gamma_{1})(\gamma_{2}+\beta_{2}-\lambda_{0}-\beta_{1})-\lambda_{1}z_{2}-z_{1}z_{2}-z_{1}\gamma_{1},\\ a_{0}≔(\lambda_{0}+\beta_{1})\lambda_{1}(\lambda_{2}+\alpha_{2})-(\gamma_{0}+\alpha_{1})\gamma_{1}(\gamma_{2}+\beta_{2}), \end{array}$$

with *z*_1_ ≔ λ_0_ + *γ*_0_ + *α*_1_ + *β*_1_ and *z*_2_ ≔ λ_2_ + *γ*_2_ + *α*_2_ + *β*_2_.

The polynomial equation admits several solutions for *R*, but only one solution corresponds to the unique steady-state *e* ∈ *C*^2^. For example, for λ_*i*_ = 1, *γ*_*i*_ = 2, *β*_*i*_ = 3, and *α*_*i*_ = 4 for all *i* the polynomial equation becomes −*R*^2^ − 131*R* − 40 = 0. This admits two solutions *R*_1_ ≔ (−3*s* − 131)/2 and *R*_2_ ≔ (3*s* − 131)/2, with $s≔\sqrt{1889}$. Substituting *R*_1_ in Eq (17) yields *e* = [*e*_1_ *e*_2_]′, with *e*_2_ < 0, so this is not a feasible solution. Substituting *R*_2_ in Eq (17) yields (all numbers are to four digit accuracy) *e* = [0.4305 0.4695]′ ∈ *C*^2^, which is the unique feasible solution. Thus, the steady-state output rate is *R*_2_ = −0.3046.

In general, Eq (16) can be transformed into a polynomial equation for *R*. The next result shows that the degree of this polynomial equation grows quickly with *n*.

**Proposition 4** *Consider the MFALK with dimension* *n* *and with* λ_*i*_ ≠ *γ*_*i*_, *α*_*i*_ ≠ 0, *β*_*i*_ ≠ 0, *for all* *i*. *Then generically* Eq (16) *may be written as* *w*(*R*) = 0, *where* *w* *is a polynomial of degree* $1+\lfloor \frac{2^{n}}{3}\rfloor$, *and with coefficients that are algebraic functions of the rates*.

We note that this exponential increase in the degree of the polynomial equation is a feature of the MFALK that does not take place in the RFM. Indeed, in the RFM the degree of the polynomial equation for the steady-state production rate grows linearly with *n*.

Let sgn(·):R→{-1,0,1} denote the sign function, i.e.

$$\begin{array}{c} \mathrm{sgn}\left(y\right)=\{\begin{array}{cc} 1, & y>0,\\ 0, & y=0,\\ -1, & y<0. \end{array} \end{array}$$

An interesting question is how does sgn(*R*) depend on the parameters. Indeed, if *R* > 0 [*R* < 0] then there is a net steady-state flow from left to right [right to left]. The next subsection describes a special case where this question can be answered rigorously.

#### 2.3.1 Bidirectional flow with no Langmuir kinetics

When *β*_*i*_ = *α*_*i*_ = 0, *i* = 1, …, *n*, i.e. a system with no internal attachments and detachments, Eq (15) becomes

$$\begin{array}{c} R=f_{i}\left(e\right),\;i=0,\ldots ,n. \end{array}$$ (18)

**Proposition 5** *Consider the case where* *α*_*i*_ = *β*_*i*_ = 0, *i* = 1, …, *n*, *and suppose that* Eq (9) *holds*. *Then*

$$\begin{array}{c} \mathrm{sgn}\left(R\right)=\mathrm{sgn}(\prod_{i=0}^{n}\lambda_{i}-\prod_{i=0}^{n}\gamma_{i}). \end{array}$$ (19)

*In particular*, *if* $\prod_{i=0}^{n}\lambda_{i}=\prod_{i=0}^{n}\gamma_{i}$ *then* *R* = 0, *and for any* *i*,

$$\begin{array}{ll} e_{i} & =\frac{\prod_{j=0}^{i-1}\lambda_{j}}{\prod_{j=0}^{i-1}\lambda_{j}+\prod_{j=0}^{i-1}\gamma_{j}}\\ & =\frac{\prod_{j=i}^{n}\gamma_{j}}{\prod_{j=i}^{n}\gamma_{j}+\prod_{j=i}^{n}\lambda_{j}}. \end{array}$$ (20)

Eq (19) means that in the case of no Langmuir kinetics the steady-state output from the right hand-side of the chain will be positive [negative] if the product of the forward rates is larger [smaller] than the product of the backward rates. In transcription and translation the steady-state flow from the right hand-side of the chain should always be positive, but in other cases, e.g. transport along microtubules, the steady state flow may be either positive or negative.

Eq (20) is also quite intuitive. It considers the case of no Langmuir kinetics and when the product of all the forward rates equals the product of all the backward rates, i.e. $\prod_{i=0}^{n}\lambda_{i}=\prod_{i=0}^{n}\gamma_{i}$. In this case the steady-state flow is zero, and the steady-state density at site *i* is the product of all the forward rates up to *i*, that is, λ_0_λ_1_ … λ_*i*−1_ normalized by the sum of two terms: the product of all the forward rates up to *i* and the product of all the backward rates up to *i*.

### 2.4 Entrainment

Assume now that some or all the rates are time-varying periodic functions with the same period *T*. This may be interpreted as a periodic excitation feeding the MFALK. Many biological processes are affected by such excitations due for example to the periodic 24h solar day or the periodic cell-cycle division process. For example, translation elongation factors, tRNAs, translation and transcription initiation factors, ATP levels, and more may change in a periodic manner and this may be modeled using periodic rates in the MFALK.

A natural question is: will the state-variables of the MFALK converge to a periodic pattern with period *T*? We will show that this is indeed so, i.e. the MFALK *entrains* to a periodic excitation in the rates. In order to understand what this means, consider a different setting, namely, using the MFALK to model traffic flow. Then the rates may correspond to traffic lights, changing in a periodic manner, and the state-variables are the density of the moving particles (cars) along different sections of the road, so entrainment corresponds to what is known as the “green wave” (see e.g. [63] and the references therein).

We say that a function *f* is *T*-periodic if *f*(*t* + *T*) = *f*(*t*) for all *t*. Assume that the λ_*i*_s, *γ*_*i*_s, *α*_*i*_s and *β*_*i*_s are uniformly bounded, non-negative, time-varying functions satisfying:

- there exists a (minimal) *T* > 0 such that all the λ_*i*_(*t*)s, *γ*_*i*_(*t*)s, *α*_*i*_(*t*)s, and *β*_*i*_(*t*)s are *T*-periodic.
- there exist *c*_1_, *c*_2_ > 0 such that at least one of the following two conditions holds for all time *t*
  $$\lambda_{i}(t)+\beta_{i+1}(t)>c_{1},\;i=0,\ldots ,n,$$ (21)
  $$\gamma_{i}(t)+\alpha_{i+1}(t)>c_{2},\;i=0,\ldots ,n.$$ (22)
- there exists *c*_3_ > 0 such that
  $$\lambda_{i}(t)+\gamma_{i+1}(t)>c_{3},\;i=0,\ldots ,n.$$ (23)

We refer to this model as the *Periodic MFALK (PMFALK)*.

**Theorem 1** *Consider the PMFALK with dimension* *n*. *There exists a unique function* $\phi \left(\cdot \right):R_{+}\to \mathrm{int}\left(C^{n}\right)$, *that is* *T*-*periodic*, *and for any* *a* ∈ *C*^*n*^ *the trajectory* *x*(*t*, *a*) *converges to* *ϕ* *as* *t* → ∞.

Thus, the PMFALK *entrains* (or phase-locks) to the periodic excitation in the parameters. In particular, this means that the output rate *R*(*t*) in Eq (3) converges to the unique *T*-periodic function:

$$\begin{array}{c} \left(\lambda_{n}\left(t\right)+\gamma_{n}\left(t\right)+\beta_{n}\left(t\right)+\alpha_{n}\left(t\right)\right)\phi_{n}\left(t\right)-\gamma_{n}\left(t\right)-\beta_{n}\left(t\right). \end{array}$$

Note that since a constant function is a periodic function for all *T* ≥ 0, Theorem 1 implies that entrainment holds also in the particular case where a *single* parameter is oscillating (with period *T* > 0), while all other parameters are constant. Note also that Corollary 1 follows from Theorem 1.

**Fig 4:**
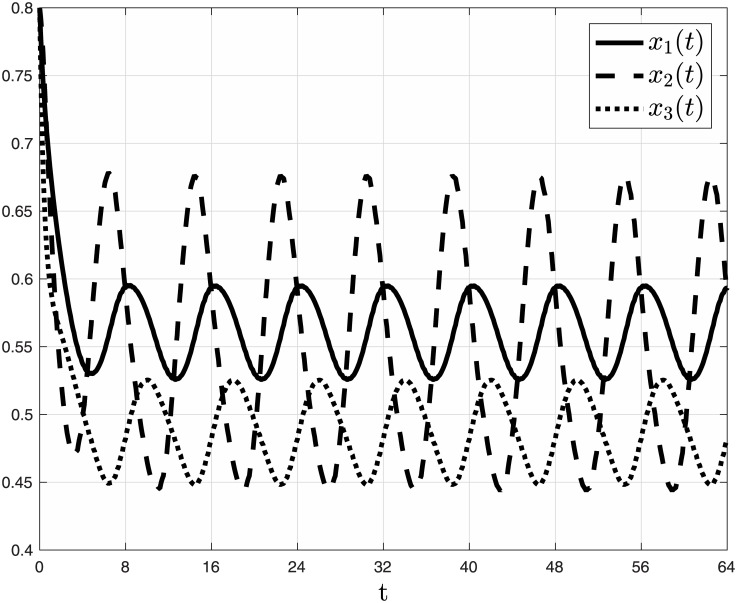
State variables *x*_1_(*t*) [solid line]; *x*_2_(*t*) [dashed line]; and *x*_3_(*t*) [dotted line] as a function of *t* in Example 3. Note that each state variable converges to a periodic function with a period *T* = 8.

**Example 3** Consider the MFALK with dimension *n* = 3, parameters: λ_0_(*t*) ≡ 1.0, λ_1_(*t*) ≡ 1.2, λ_2_(*t*) = 1 + 0.5sin(*πt*/4), λ_3_(*t*) ≡ 0.9, *γ*_0_(*t*) ≡ 0.4, *γ*_1_(*t*) = 0.4(1 + sin((*πt*/4) + 1/2)), *γ*_2_(*t*) ≡ 0.25, *γ*_3_(*t*) ≡ 0.45, *α*_1_(*t*) ≡ 0, *α*_2_(*t*) ≡ 0.05, *α*_3_(*t*) ≡ 0, *β*_1_(*t*) ≡ 0, *β*_2_(*t*) = 0.05(1 + sin((*πt*/2) + 1/4)), *β*_3_(*t*) ≡ 0, and initial condition *x*(0) = [0.8 0.8 0.8]′. Note that all the rates here are periodic, with a minimal common period *T* = 8. Fig 4 depicts *x*_*i*_(*t*), *i* = 1, 2, 3, as a function of *t*. It may be seen that each state variable converges to a periodic function with period *T* = 8.

Since the MFALK is a mean-field approximation of ASEP with Langmuir kinetics, a natural question is does ASEP with Langmuir kinetics entrains as well (in some stochastic manner)? The following example addresses this question using Monte Carlo simulations.

**Example 4** Consider ASEP with Langmuir kinetics with *N* = 8 sites and hopping rates:

- $\lambda_{1}\left(t\right)=\frac{1}{2}+\frac{1}{4}\sin\left(\frac{2\pi t}{T}\right)$, and $\lambda_{i}\left(t\right)\equiv \frac{1}{2}$, for all *i* ≠ 1,
- *γ*_*i*_(*t*)≡0, *i* = 0, …, 8,
- *α*_*i*_(*t*)≡0, *i* = 1, …, 8,
- $\beta_{6}\left(t\right)=\frac{1}{5}+\frac{1}{10}\cos\left(\frac{2\pi t}{T}\right)$, and *β*_*i*_(*t*) ≡ 0, *i* ≠ 6,

where *T* = 1*E*7. Note that all these rates are periodic with a common minimal period *T*. When simulating ASEP with Langmuir kinetics, these rates are used to determine the next event time (i.e., in this example, the next forward hopping event or the next attachment event). Given a corresponding rate *r*(*t*) at time *t*, the next event time is *t* + *p*(*r*(*t*)), where *p*(*r*(*t*)) is a random variable drawn from the exponential distribution with mean *r*(*t*).

**Fig 5:**
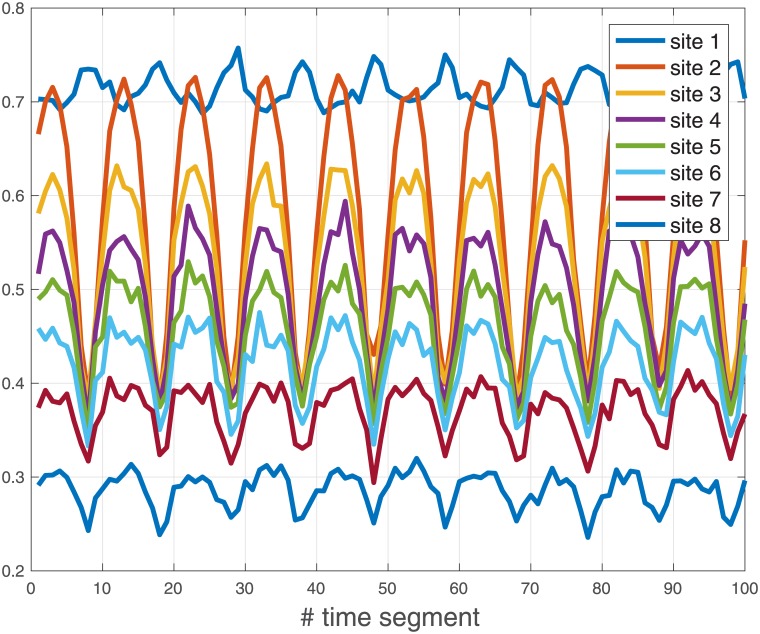
The average occupancies of ASEP with Langmuir kinetics in Example 4. Note that each average occupancy converges to a periodic function with a period *T* = 10 segments.

We ran MATLAB simulations of this process for 1*E*8 time ticks. Fig 5 depicts the average occupancies per site. Each data point in the figure (i.e. each segment) is the average over 1*E*6 consecutive occupancies (here of course the occupancies are either 0 or 1). For example, the value depicted at time segment 1 is the average occupancy in the time interval [0, 999999]. Note that since *T* = 1*E*7 time ticks, the period *T* is equal to 10 segments. It may be seen that all the average occupancies indeed entrain to the periodic excitation. In particular, they are periodic (up to the noise induced by the stochastic process) with a period of 10 segments. Thus, our simulations do suggest that some form of entrainment also takes place in ASEP with Langmuir Kinetics.

### 2.5 Strong monotonicity

Recall that a proper cone $K\subseteq R^{n}$ defines a partial ordering in $R^{n}$ as follows. For two vectors $a,b\in R^{n}$, we write *a* ≤ *b* if (*b* − *a*) ∈ *K*; *a* < *b* if *a* ≤ *b* and *a* ≠ *b*; and *a* ≪ *b* if (*b* − *a*) ∈ int(*K*). The system $\dot{y}=f\left(y\right)$ is called *monotone* if *a* ≤ *b* implies that *y*(*t*, *a*) ≤ *y*(*t*, *b*) for all *t* ≥ 0. In other words, the flow preserves the partial ordering [64]. It is called *strongly monotone* if *a* < *b* implies that *y*(*t*, *a*) ≪ *y*(*t*, *b*) for all *t* > 0.

From here on we consider the particular case where the cone is $K≔R_{+}^{n}$. Then *a* ≤ *b* if *a*_*i*_ ≤ *b*_*i*_ for all *i*, and *a* ≪ *b* if *a*_*i*_ < *b*_*i*_ for all *i*. A system that is monotone with respect to this partial ordering is called *cooperative*.

**Proposition 6** *For any* *a*, *b* ∈ *C*^*n*^, *with* *a* ≤ *b*, *the solutions of the MFALK satisfy*

$$\begin{array}{c} x(t,a)\leq x(t,b),\;for\;all\;t\geq 0. \end{array}$$ (24)

*Furthermore*, *if* Eq (9) *holds then for any* *a* < *b*

$$\begin{array}{c} x(t,a)\ll x(t,b),\;for\;all\;t>0. \end{array}$$ (25)

To explain this, consider two initial densities *a* and *b* with *a*_*i*_ ≤ *b*_*i*_ for all *i*, that is, *b* corresponds to a larger or equal density at each site. Then the trajectories *x*(*t*, *a*) and *x*(*t*, *b*) emanating from these initial conditions continue to satisfy the same relationship between the densities, namely, *x*_*i*_(*t*, *a*) ≤ *x*_*i*_(*t*, *b*), for all *i* and for all time *t* ≥ 0.

The MFALK is thus a *strongly cooperative tridiagonal system* (SCTS) on int(*C*^*n*^). Some of the properties deduced above using contraction theory can also be deduced using this property [65].

**Remark 1** *Suppose that we augment the MFALK into a model of* *n* + 1 *ODEs in* *n* + 1 *state-variables by adding to it the equation*:

$$\begin{array}{ll} {\dot{x}}_{n+1} & =-\lambda_{0}(1-x_{1})-\gamma_{0}x_{1}-\beta_{1}(1-x_{1})+\alpha_{1}x_{1}\\ & -\gamma_{n}(1-x_{n})+\lambda_{n}x_{n}-\beta_{n}(1-x_{n})+\alpha_{n}x_{n}\\ & -\sum_{i=2}^{n-1}\left(\beta_{i}(1-x_{i})-\alpha_{i}x_{i}\right). \end{array}$$

*that is*, ${\dot{x}}_{n+1}=-\sum_{i=1}^{n}{\dot{x}}_{i}$ *(see* Eq (2) *)*. *Let* $\tilde{x}$ *denote the vector of the* *n* + 1 *state-variables*. *Clearly*, *this augmented model admits a first integral* $H\left(\tilde{x}\left(t\right)\right)≔\sum_{i=1}^{n+1}{\tilde{x}}_{i}\left(t\right)$, *i.e. a property that is preserved for all* *t* ≥ 0. *Also*, *for any initial condition in* $\tilde{x}\left(0\right)\in C^{n}\times R_{+}$, *all the state-variables remain bounded, as the first* *n* *state-variables remain in* *C*^*n*^ and ${\tilde{x}}_{n+1}\left(t\right)=H\left(\tilde{x}\left(0\right)\right)-\sum_{i=1}^{n}{\tilde{x}}_{i}\left(t\right)$ for all *t* ≥ 0. *It is straightforward to verify that the augmented system is a cooperative system, and that if* Eq (9) *holds then it is a SCTS. SCTSs that admit a non-trivial first integral have many desirable properties (see, e.g.* [66]*)*.

### 2.6 Effect of attachment and detachment

One may perhaps expect that detachment from a jammed site may increase the total flow by reducing congestion. The next result shows that this is not so. Detachment always decreases the steady-state output rate *R*. Similarly, attachment always increases *R*.

**Proposition 7** *Consider a MFALK with dimension* *n*. *Suppose that the conditions in Proposition 3 hold*. *Then* $\frac{\partial e_{i}}{\partial \alpha_{j}}<0$, *and* $\frac{\partial e_{i}}{\partial \beta_{j}}>0$, *for all* *i*, *j*. *Also*, $\frac{\partial R}{\partial \alpha_{j}}<0$, *and* $\frac{\partial R}{\partial \beta_{j}}>0$ *for all* *j* = 0, 1, …, *n* − 1.

This means that an increase in any of the detachment [attachment] rates decreases [increases] the steady-state density in all the sites. Also, an increase in any of the internal detachment [attachment] rates decreases [increases] the steady-state output rate. The next example demonstrates this.

**Fig 6:**
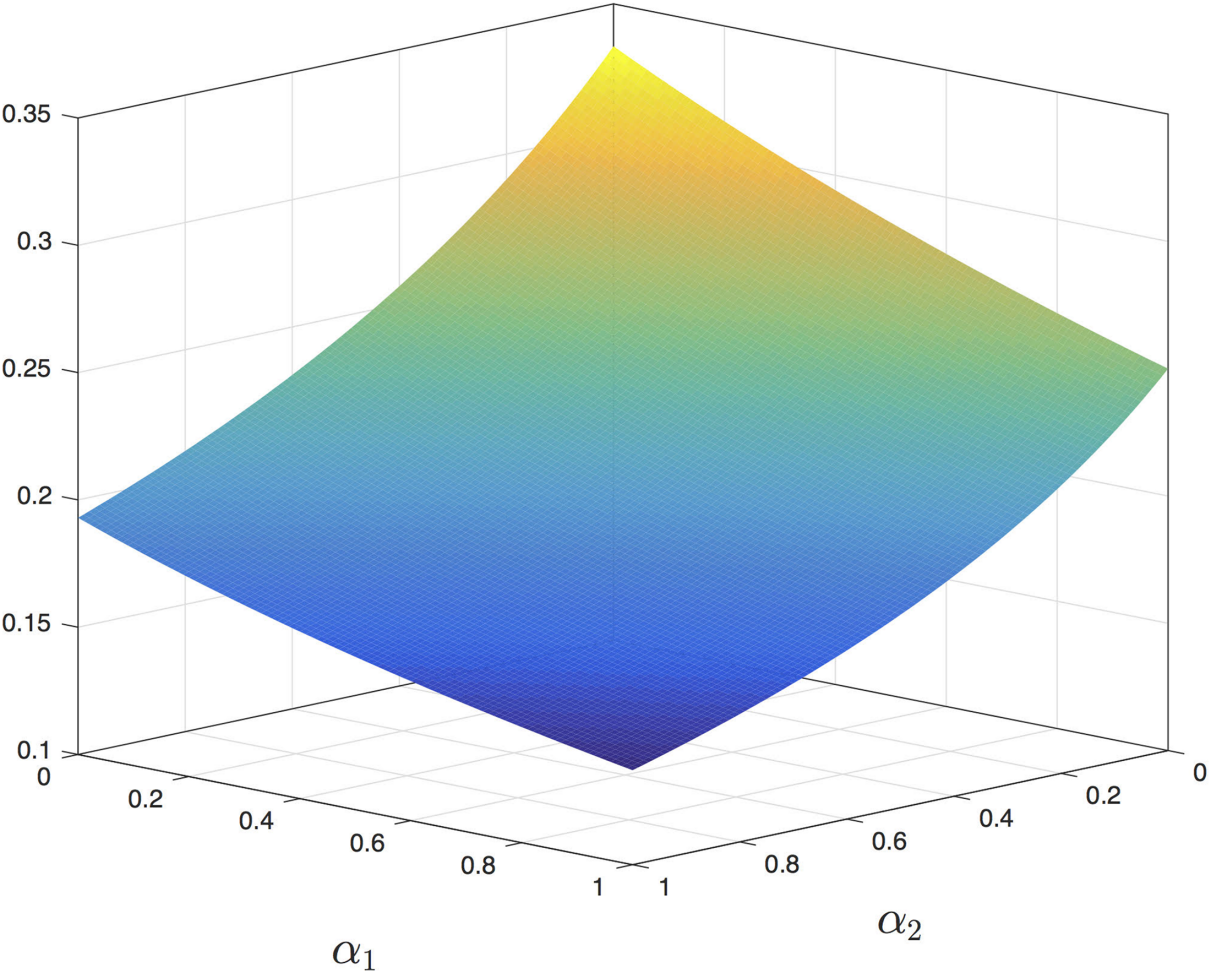
*R* as a function of *α*_1_ ∈ [0, 1] and *α*_2_ ∈ [0, 1] for the MFALK in Example 5. It may be seen that *R* decreases with both *α*_1_ and *α*_2_.

**Example 5** Consider the MFALK with *n* = 3, λ_*i*_ = 1, *γ*_*i*_ = 0, *i* = 0, 1, 2, 3, *β*_*i*_ = *α*_3_ = 0, *i* = 1, 2, 3. Fig 6 depicts *R* as a function of *α*_1_ ∈ [0, 1] and *α*_2_ ∈ [0, 1]. It may be seen that *R* decreases with both *α*_1_ and *α*_2_.

We note that the analytical results in Proposition 7 agree well with the simulation results obtained using a TASEP model for translation that included alternative initiation along the mRNA and ribosome drop-off [67].

The next section describes an application of the MFALK to a biological process.

## 3 An application: Modeling mRNA translation with ribosome drop off

It is believed that during mRNA translation ribosome movement is unidirectional from the 5’ end to the 3’ end, and that ribosomes do not enter in the middle of the coding regions. However, ribosomes can detach from various sites along the mRNA molecule due to collisions between ribosomes, for example. This is known as ribosome drop off.

As mentioned in the introduction, ribosome drop off has been the topic of numerous studies [12, 13, 44–51, 68]. It was suggested that in some cases ribosome drop off is important for proof reading [54], and also that ribosome stalling/abortion plays a role in translational regulation (e.g. see [67, 69]).

It is clear that ribosome abortion has drawbacks. Indeed, translation is the most energetically consuming process in the cell, and abortion results in truncated, non-functional and possibly deleterious proteins. It is believed that transcripts undergo evolutionary selection to minimize abortion and/or its energetic cost [12, 48, 49, 51–53]. Nevertheless, there seems to be a certain minimal abortion rate even in non-stressed conditions [13, 68]. This basal value was estimated (see more details below) to be of the order or 10^−4^ − 10^−3^ abortion events per codon in *E. coli*. In other words, at each codon one out of 1,000–10,000 decoding ribosomes aborts. This value is non-negligible. If we consider a drop-off rate of 4 × 10^−4^ per codon along a coding region of 300 codons (approximately the average coding region length in *E. coli*) then on average, around 10 out of every 100 ribosomes will fail to complete translation of the mRNA.

To model translation with ribosome drop off, we use the MFALK with *γ*_*i*_ = 0 (i.e. no backwards motion) and *β*_*i*_ = 0 (i.e. no attachment to internal sites along the chain) for all *i*. Changing the values of the *α*_*i*_s allows to model and analyze the effect of ribosome drop off at different sites along the mRNA molecule. We assume that

$$\begin{array}{c} \lambda_{i}>0,\;\text{for}\;\text{all}\;i, \end{array}$$ (26)

as otherwise the chain decouples into two smaller, disconnected chains. Note that Eq (26) implies that the conditions in Proposition 3 hold, so the model is SO on *C*^*n*^ w.r.t. the *L*_1_ norm, and thus admits a unique globally asymptotically stable steady-state *e* ∈ int(*C*^*n*^).

**Fig 7:**
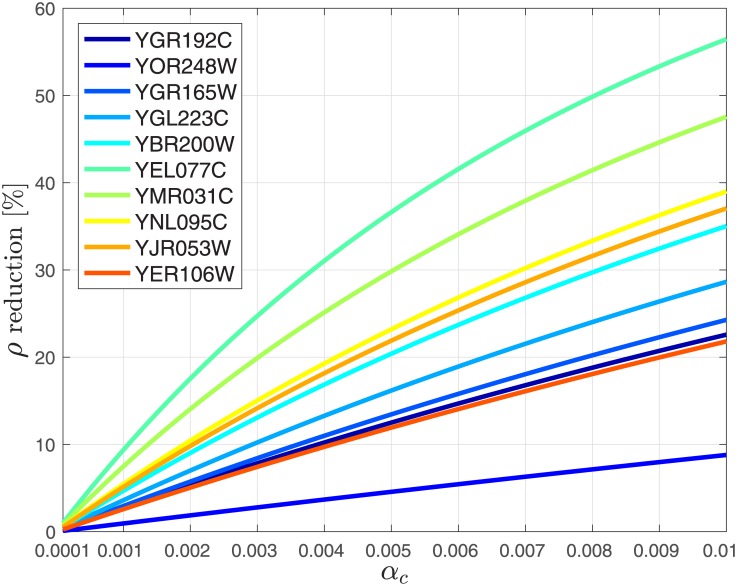
Reduction in percent in the steady-state mean density *ρ* as a function of *α*_*c*_ ∈ [10^−4^, 10^−2^] for 10 *S. cerevisiae* genes.

**Fig 8:**
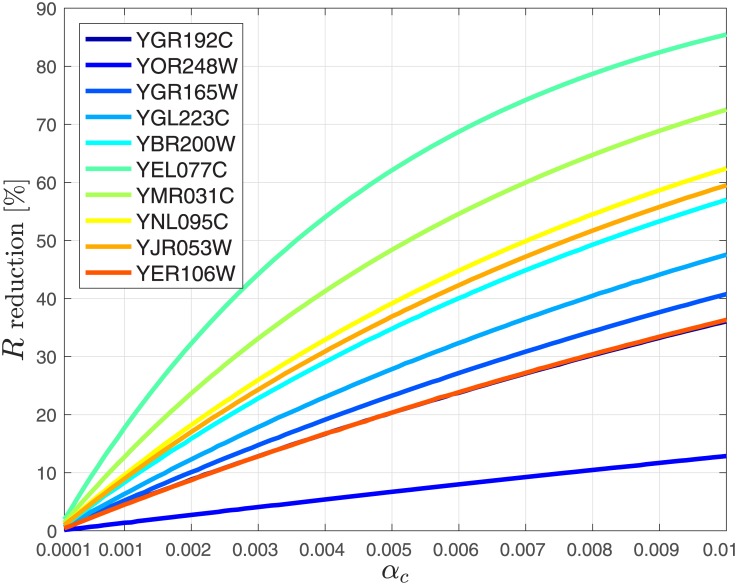
Reduction in percent in the steady-state output rate (production rate) *R* as a function of *α*_*c*_ ∈ [10^−4^, 10^−2^] for 10 *S. cerevisiae* genes.

We study the effect of ribosome drop off on the steady-state protein production rate and the steady-state ribosome density using real biological data. To this end, we first considered 10 *S. cerevisiae* genes (see Figs 7 and 8) with various mRNA levels (all genes were sorted according to their mRNA levels and 10 genes were uniformly sampled from the list). Similarly to the approach used in [28], we divided the mRNAs related to these genes to non-overlapping pieces. The first piece includes the first 9 codons that are related to various stages of initiation [53]. The other pieces include 10 non-overlapping codons each, except for the last one that includes between 5 and 15 codons.

To model translation dynamics in these mRNAs using MFALK, we model every piece of mRNA as a site. We estimated the elongation rates λ_*i*_ at each site using ribo-seq data for the codon decoding rates [70], normalized so that the median elongation rate of all *S. cerevisiae* mRNAs becomes 6.4 codons per second [71]. We first applied a filter that considers the biases and traffic jams in ribo-seq to infer for each of the 61 codons a typical decoding time; these times are normalized to get an elongation rate of 6.4 codons/sec [71]. The site rate is (site time)^−1^, where site time is the sum over the decoding times of all the codons in the piece of mRNA corresponding to this site. These rates thus depend on various factors including availability of tRNA molecules, amino acids, Aminoacyl tRNA synthetase activity and concentration, and local mRNA folding [5, 53, 70].

The initiation rate λ_0_ (corresponding to the first piece) was inferred based on the fact that the ribosome density (and the translation rate) is proportional to the initiation rate when it is rate limiting [28, 30]. Thus, since in endogenous genes initiation is typically rate limiting the ribosome density should roughly be proportional to the initiation rate. We then normalized the initiation rates such that their mean match the measured/known initiation rate (0.8 initiations per second) [10].

We analyzed the effect of uniform ribosome drop off with a rate in the range of 10^−5^ to 10^−3^ per codon. This corresponds to *α*_1_ = ⋯ = *α*_*n*_ ≔ *α*_*c*_, i.e., all the *α*_*i*_s are equal, and *α*_*c*_ denotes their common value. Since we assumed 10 codons per site, *α*_*c*_ values range from 10^−4^ to 10^−2^ (ten times the rate associated with a single codon). This makes sense as in the MFALK the level of occupancy in a site may also be interpreted as the probability to see a ribosome in this site.

Let

$$\begin{array}{c} \rho ≔\frac{\sum_{i=1}^{n}e_{i}}{n}, \end{array}$$

denote the steady-state mean ribosomal density. Figs 7 and 8 depict the reduction in percentage in *ρ* and *R*, respectively, as a function of *α*_*c*_ ∈ [10^−4^, 10^−2^]. In these figures the genes in the legends are sorted according to their expression levels: the gene at the top (YGR192C) has the highest mRNA levels while the gene at the bottom (YER106W) has the lowest levels. It may be seen that as the drop off (detachment) rate *α*_*c*_ increases from 10^−4^ to 10^−2^, *ρ* decreases by about 30%, and *R* decreases by about 50%. This demonstrates the significant ramifications that ribosomal drop off is expected to have on translation and the importance of modeling drop off. It may also be observed that in general for mRNAs with higher expression levels (i.e. mRNAs with higher copy number in the cell) the reduction in both the steady-state production rate and mean density due to drop off is lower as compared to the reduction for mRNAs with low copy number.

**Fig 9:**
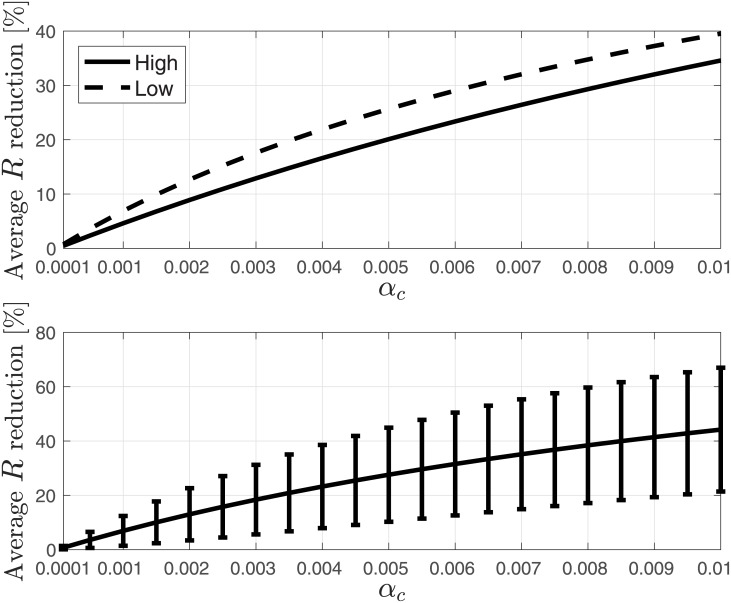
Average reduction in percent in the steady-state output rate (production rate) *R* as a function of *α*_*c*_ ∈ [10^−4^, 10^−2^]. Upper figure: reduction over most expressed (top 20%) *S. cerevisiae* genes (solid-line), and over least expressed (bottom 20%) *S. cerevisiae* genes (dashed-line). Lower figure: reduction over all *S. cerevisiae* genes. Also shown are the variances as error bars.

**Fig 10:**
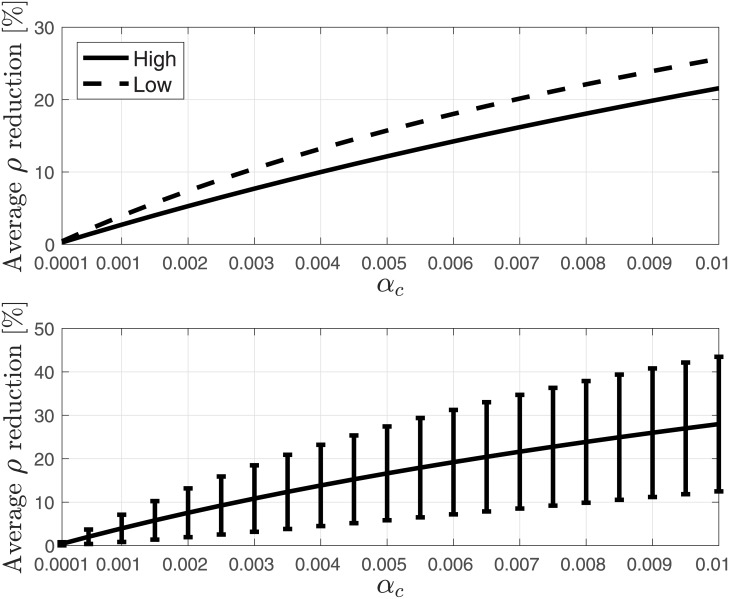
Average reduction in percent in the steady-state mean density *ρ* as a function of *α*_*c*_ ∈ [10^−4^, 10^−2^]. Upper figure: reduction over most expressed (top 20%) *S. cerevisiae* genes (solid-line), and over least expressed (bottom 20%) *S. cerevisiae* genes (dashed-line). Lower figure: reduction over all *S. cerevisiae* genes. Also shown are the variances as error bars.

We next evaluated the reduction due to drop off over: 1) all 6310 protein-encoding *S. cerevisiae* genes; 2) most expressed *S. cerevisiae* genes (top 20%); and 3) least expressed *S. cerevisiae* genes (bottom 20%). The average reduction in *R* and *ρ* over these three sets of genes are depicted in Figs 9 and 10, respectively. It may be noticed that the average reduction over the highly expressed genes is lower than the average reduction over the lowly expressed genes. It is possible that this is related to stronger evolutionary selection for lower drop off rates in genes with higher mRNA levels. Indeed, highly expressed genes “consume” more ribosomes (due to higher mRNA levels), so a given (per-mRNA) drop off rate is expected to be more deleterious to the cell, and a mutation which decreases the drop off rate in such genes has a higher probability of fixation.

## 4 Discussion

In many important processes biological “particles” move along some kind of a one-dimensional “track”. Examples include gene transcription and translation, cellular transport, and more. The flow can be either bidirectional (as in the case of transcription) or unidirectional (as in the case of translation elongation), with the possibility of both attachment and detachment of particles at different sites along the track. For example, motor proteins like kinesin and dynein that move along a certain microtubule may detach and attach to an overlapping microtubule.

To rigorously model and analyze such processes, we introduced a new deterministic mathematical model that can be derived as a dynamic mean-field approximation of ASEP with Langmuir kinetics, called the MFALK. Our main results show that the MFALK is a monotone and contractive dynamical system. This implies that it admits a globally asymptotically unique steady-state, and that it entrains to periodic excitations (with a common period *T* > 0) in any of its rates, i.e. the densities along the chain, as well as the output rate, converge to a unique period solutions with period *T*.

It is important to note that several known models are special cases of the MFALK. These include for example the RFM [28], the model used in [72] for DNA transcription, the model used in [73] for the various hydrolysis products of a molecular motor, and the model of phosphorelays in [74] (although in the latter model the occupancy levels are normalized differently).

Topics for further research include the following. In the RFM, it has been shown that the steady-state production rate is related to the maximal eigenvalue of a certain non-negative, symmetric tridiagonal matrix with elements that are functions of the RFM rates, i.e. the λ_*i*_s [35]. This implies that the mapping (λ_0_, …, λ_*n*_) → *R* is strictly concave, and that sensitivity analysis of *R* is an eigenvalue sensitivity problem [36]. Similar results have also been derived for the ribosome flow model on a ring (RFMR) [75] which is a mean-field approximation of TASEP with periodic boundary conditions. An interesting research topic is whether *R* = *R*(λ_0_, …, λ_*n*_, *γ*_0_, …, *γ*_*n*_, *α*_1_, …, *α*_*n*_, *β*_1_, …, *β*_*n*_) in the MFALK can also be described using such a linear-algebraic approach.

The application of the MFALK to model ribosome drop off suggests an interesting direction for further study, namely, how to design genes that minimize the drop off rate.

Another research direction is motivated by the fact that many of the transport phenomena that can be modeled using the MFALK do not take place in isolation. For example, many mRNA molecules are translated in parallel in the cell. Thus, a natural next step is to study networks of interconnected MFALKs. In this context, ribosome drop off may perhaps increase the total production rate in the entire system, as it allows ribosomes to detach from slow sites, enter the pool of free ribosomes, and then attach to the initiation sites of other, less crowded, mRNA molecules. However, drop off still incurs the biological “cost” associated with the synthesis of a chain of amino-acids that is only a part of the desired protein. The fact that the MFALK is contractive may prove useful in analyzing networks of MFALKs, as there exist interesting results proving the overall contractivity of a network based on contractivity of the subsystems and their couplings (see, e.g. [76, 77]).

Finally, another interesting topic for further research is studying the effect of controlled detachment rates on the formation of traffic jams. Indeed, it is known that kinesin-family motor proteins are more susceptible to dissociation when their path is blocked [14, 15].

## Supporting information

S1 File This is the proof file. This file includes the proofs of all the theorems in the paper.
(PDF)
