# Supplementary material for: A deterministic mathematical model for bidirectional excluded flow with Langmuir kinetics
Source: PLoS One. 2017 Aug 23;12(8):e0182178. doi: 10.1371/journal.pone.0182178 (PMC5568237; doi:10.1371/journal.pone.0182178)
Supplement: S1 File — This file includes the proofs of all the theorems in the paper. (PDF) [file pone.0182178.s001.pdf]

Appendix: A Deterministic Mathematical Model for Bidirectional Excluded Flow with Langmuir Kinetics

Yoram Zarai, Michael Margaliot and Tamir Tuller

A. PROOFS

We begin by discussing some symmetry properties of the MFALK.

A. *Symmetry*

The MFALK enjoys two symmetries that will be useful later on. First, let $z_i(t) := 1-x_i(t)$, $i = 1, \dots, n$. In other words, $z_i(t)$ is the amount of "free space" at site i at time t. Then using (1) yields

$$\begin{aligned}
\dot{z}_1 &= \gamma_0(1-z_1) + \lambda_1 z_2(1-z_1) + \alpha_1(1-z_1) - \gamma_1 z_1(1-z_2) - \lambda_0 z_1 - \beta_1 z_1,\\
\dot{z}_2 &= \gamma_1 z_1(1-z_2) + \lambda_2 z_3(1-z_2) + \alpha_2(1-z_2) - \gamma_2 z_2(1-z_3) - \lambda_1 z_2(1-z_1) - \beta_2 z_2,\\
&\vdots\\
\dot{z}_n &= \gamma_{n-1} z_{n-1}(1-z_n) + \lambda_n(1-z_n) + \alpha_n(1-z_n) - \gamma_n z_n - \lambda_{n-1} z_n(1-z_{n-1}) - \beta_n z_n.
\end{aligned} \tag{A.1}$$

This is just the MFALK (1), but with the parameters permuted as follows: $\lambda_k \to \gamma_k$, $\gamma_k \to \lambda_k$, $\beta_k \to \alpha_k$, and $\alpha_k \to \beta_k$ for all k. The symmetry here follows from the fact that we can replace the roles of the forward and backward flows in the MFALK.

Next, let $y_i(t) := 1 - x_{n+1-i}(t)$, $i = 1, \dots, n$. In other words, $y_i(t)$ is the amount of "free space" at site $n+1-i$ at time t. Then using (1) yields

$$\begin{aligned}
\dot{y}_1 &= \lambda_n(1-y_1) + \gamma_{n-1} y_2(1-y_1) + \alpha_n(1-y_1) - \lambda_{n-1} y_1(1-y_2) - \gamma_n y_1 - \beta_n y_1,\\
\dot{y}_2 &= \lambda_{n-1} y_1(1-y_2) + \gamma_{n-2} y_3(1-y_2) + \alpha_{n-1}(1-y_2) - \lambda_{n-2} y_2(1-y_3) - \gamma_{n-1} y_2(1-y_1) - \beta_{n-1} y_2,\\
&\vdots\\
\dot{y}_n &= \lambda_1 y_{n-1}(1-y_n) + \gamma_0(1-y_n) + \alpha_1(1-y_n) - \lambda_0 y_n - \gamma_1 y_n(1-y_{n-1}) - \beta_1 y_n.
\end{aligned} \tag{A.2}$$

This is just the MFALK (1), but with the parameters permuted as follows: $\lambda_k \to \lambda_{n-k}$, $\gamma_k \to \gamma_{n-k}$, $\beta_k \to \alpha_{n+1-k}$, and $\alpha_k \to \beta_{n+1-k}$ for all k. Note that (A.1) is simply (A.2) with the variable renaming $z_i \to y_{n+1-i}$, $i = 1, \dots, n$.

Both symmetries are reminiscent of the *particle-hole symmetry* in ASEP [2], [3]: the basic idea is that the progression of a particle from left to right is also the progression of a "hole" from right to left.

The research of YZ is partially supported by the Edmond J. Safra Center for Bioinformatics at Tel Aviv University. The research of MM and TT is partially supported by research grants from the Israeli Ministry of Science and Technology, and the US-Israel Binational Science Foundation. The research of MM is also supported by a research grant from the Israel Science Foundation.

Y. Zarai is with the School of Electrical Engineering, Tel-Aviv University, Tel-Aviv 69978, Israel. E-mail: yoramzar@mail.tau.ac.il
M. Margaliot is with the School of Electrical Engineering and the Sagol School of Neuroscience, Tel-Aviv University, Tel-Aviv 69978, Israel. E-mail: michaelm@eng.tau.ac.il
T. Tuller is with the Dept. of Biomedical Engineering and the Sagol School of Neuroscience, Tel-Aviv University, Tel-Aviv 69978, Israel. E-mail: tamirtul@post.tau.ac.il

Proof of Proposition 1. If (4) holds then the MFALK satisfies property (**BR**) in [4], and [4, Lemma 1] implies (6). If (5) holds then (A.1) satisfies property (**BR**) in [4], and this implies (6). □

Proof of Proposition (2). Write the MFALK as $\dot{x} = f(x)$. A calculation shows that the Jacobian matrix $J(x) := \frac{\partial f}{\partial x}(x)$ satisfies $J(x) = L(x) + P$, where $L(x)$ is the matrix

$$\begin{bmatrix} -\lambda_1(1-x_2)-\gamma_1 x_2 & \lambda_1 x_1+\gamma_1(1-x_1) & \dots & 0 \\ \lambda_1(1-x_2)+\gamma_1 x_2 & -\lambda_1 x_1-\gamma_1(1-x_1)-\lambda_2(1-x_3)-\gamma_2 x_3 & \dots & 0 \\ 0 & \lambda_2(1-x_3)+\gamma_2 x_3 & \dots & 0 \\ & & \vdots & \\ 0 & 0 & \dots & 0 \\ 0 & 0 & \dots & \lambda_{n-1}x_{n-1}+\gamma_{n-1}(1-x_{n-1}) \\ 0 & 0 & \dots & -\lambda_{n-1}x_{n-1}-\gamma_{n-1}(1-x_{n-1}) \end{bmatrix}, \tag{A.3}$$

and P is the diagonal matrix

$$P = \operatorname{diag}(-\lambda_0-\gamma_0-\alpha_1-\beta_1, -\alpha_2-\beta_2, \dots, -\alpha_{n-1}-\beta_{n-1}, \lambda_n-\gamma_n-\alpha_n-\beta_n). \tag{A.4}$$

Note that $L(x)$ is tridiagonal and Metzler (i.e, every off-diagonal entry is non-negative) for any $x \in C^n$.

Recall that the matrix measure $\mu_1 : \mathbb{R}^{n\times n} \to \mathbb{R}$ induced by the L_1 norm is $\mu_1(A) = \max\{c_1(A), \dots, c_n(A)\}$, where $c_i(A)$ is the sum of the elements in column i of A with off-diagonal elements taken with absolute value [10]. For the Jacobian J of the MFALK, $\mu_1(J(x)) = \eta$ for all $x \in C^n$. It is well-known (see, e.g., [1]) that this implies (7). □

Proof of Proposition (3). For $\zeta \in [0, 1/2]$, let

$$C^n_\zeta := \{x \in C^n : \zeta \le x_i \le 1-\zeta,\ i = 1, \dots, n\}.$$

Note that $C^n_0 = C^n$, and that C^n_ζ is a strict subcube of C^n for all $\zeta \in (0, 1/2]$. By Proposition 1, for any $\tau > 0$ there exists $\zeta = \zeta(\tau) \in (0, 1/2)$, with $\zeta(\tau) \to 0$ as $\tau \to 0$, such that

$$x(t+\tau, a) \in C^n_\zeta, \quad \text{for all } t \ge 0 \text{ and all } a \in C^n. \tag{A.5}$$

For any $x \in C^n_\zeta$ every entry L_{ij} on the sub- and super-diagonal of L in (A.3) satisfies $L_{ij} \ge \zeta s$, where $s := \min_{1\le i\le n-1}\{\lambda_i+\gamma_i\} > 0$. Combining this with [4, Theorem 4] implies that for any $\zeta \in (0, 1/2]$ there exists $\varepsilon = \varepsilon(\zeta) > 0$, and a diagonal matrix $D = \operatorname{diag}(1, q_1, q_1 q_2, \dots, q_1 q_2 \dots q_{n-1})$, with $q_i = q_i(\varepsilon) > 0$, such that the MFALK is contractive on C^n_ζ with respect to (w.r.t.) the scaled L_1 norm defined by $|z|_{1,D} := |Dz|_1$. Furthermore, we can choose ε such that $\varepsilon(\zeta) \to 0$ as $\zeta \to 0$, and $D(\varepsilon) \to I$ as $\varepsilon \to 0$. Now Thm. 1 in [5] implies that the MFALK is contractive after a small overshoot and short transient (SOST). Prop. 4 in [5] implies that for the MFALK SOST is equivalent to SO, and this completes the proof. □

Proof of Proposition (4). We begin by recursively defining two sequences. For all integers $i \ge 1$, let

$$\begin{aligned} u_{i+1} &= 1 + \ell_1 + \ell_2 + \dots + \ell_i, \\ \ell_{i+1} &= u_i + \ell_1 + \ell_2 + \dots + \ell_{i-1}. \end{aligned} \tag{A.6}$$

with initial conditions $u_0 = u_1 = 1$, $\ell_0 = 0$, and $\ell_1 = 1$. We claim that for $k = 0, 1, \dots, n-1$, the steady-state density in site $n-k$ is generically the ratio of two polynomials in R:

$$e_{n-k} = \frac{p_k(R)}{q_k(R)}, \quad \text{with } \deg(p_k(R)) = u_k,\ \deg(q_k(R)) = \ell_k. \tag{A.7}$$

We prove this by induction on k. By (16), $e_n = aR + b$, with $a := (\lambda_n+\gamma_n+\beta_n+\alpha_n)^{-1}$ and $b := (\gamma_n+\beta_n)a$,

and this proves (A.7) for $k=0$. Using (16) again yields

$$\begin{aligned}e_{n-1} &= \frac{R+\gamma_{n-1}e_n}{\lambda_{n-1}(1-e_n)+\gamma_{n-1}e_n}\\ &= \frac{R+\gamma_{n-1}(aR+b)}{\lambda_{n-1}+(\gamma_{n-1}-\lambda_{n-1})(aR+b)},\end{aligned}$$

and this proves (A.7) for $k=1$. Now assume that there exists $s\geq 2$ such that (A.7) holds for $k=0,1,\dots,s-1$. By (16),

$$e_{n-s}=\frac{R+\gamma_{n-s}e_{n-s+1}-g_{n-s+1}(e_{n-s+1})-g_{n-s+2}(e_{n-s+2})-\dots-g_{n-1}(e_{n-1})}{\lambda_{n-s}(1-e_{n-s+1})+\gamma_{n-s}e_{n-s+1}},$$

and applying (12) and the induction hypothesis yields

$$e_{n-s}=\frac{R+\gamma_{n-s}\frac{p_{s-1}}{q_{s-1}}+(\beta_{n-s+1}+\alpha_{n-s+1})\frac{p_{s-1}}{q_{s-1}}+(\beta_{n-s+2}+\alpha_{n-s+2})\frac{p_{s-2}}{q_{s-2}}+\dots+(\beta_{n-1}+\alpha_{n-1})\frac{p_1}{q_1}+c}{\lambda_{n-s}+(\gamma_{n-s}-\lambda_{n-s})\frac{p_{s-1}}{q_{s-1}}},$$

where $c:=-\beta_{n-s+1}-\dots-\beta_{n-1}$. Multiplying the numerator and the denominator by $q_1\dots q_{s-1}$ yields $e_{n-s}=p_s/q_s$, where

$$\begin{aligned}\deg(p_s) &= \max\{1+\deg(q_1\dots q_{s-1}),\deg(p_{s-1}q_1\dots q_{s-2}),\dots,\deg(p_1q_2\dots q_{s-1})\},\\ \deg(q_s) &= \max\{\deg(q_1\dots q_{s-1}),\deg(p_{s-1}q_1\dots q_{s-2})\}.\end{aligned}$$

By the induction hypothesis,

$$\begin{aligned}\deg(p_s) &= \max\{1+\ell_1+\dots+\ell_{s-1},u_{s-1}+\ell_1+\dots+\ell_{s-2},\dots,u_1+\ell_2+\dots+\ell_{s-1}\},\\ \deg(q_s) &= \max\{\ell_1+\dots+\ell_{s-1},u_{s-1}+\ell_1+\dots+\ell_{s-2}\}.\end{aligned} \tag{A.8}$$

It is straightforward to prove that (A.6) implies that

$$\ell_i\leq u_i\leq \ell_i+1,\quad i=0,1,2,\dots. \tag{A.9}$$

Combining this with (A.8) yields $\deg(p_s)=1+\ell_1+\dots+\ell_{s-1}$, and $\deg(q_s)=u_{s-1}+\ell_1+\dots+\ell_{s-2}$. Thus, $\deg(p_s)=u_s$ and $\deg(q_s)=\ell_s$, and this completes the inductive proof of (A.7). In particular, (A.7) yields

$$e_1=\frac{p_{n-1}(R)}{q_{n-1}(R)}, \tag{A.10}$$

with $\deg(p_{n-1}(R))=u_{n-1},\ \deg(q_{n-1}(R))=\ell_{n-1}$. Substituting this in the last equation of (16) yields

$$v\frac{p_{n-1}}{q_{n-1}}=z-R+\sum_{j=2}^{n-1}g_j(e_j),$$

where $v:=\lambda_0+\gamma_0+\beta_1+\alpha_1$, and $z:=\lambda_0+\beta_1$. Arguing as above shows that this is a polynomial equation of the form $w(R)=0$, with $\deg(w)=1+\ell_1+\dots+\ell_{n-1}=u_n$. It is straightforward to prove by induction that (A.6) implies that

$$u_k=1+\left\lfloor\frac{2^k}{3}\right\rfloor,\quad \ell_k=\frac{2^k-(-1)^k}{3},$$

(we note in passing that the latter sequence is known as the Jacobsthal sequence [8]), and this completes the proof of Proposition 4. □

Proof of Proposition 5. We begin by proving that $R>0$ implies that $\prod_{i=0}^n\lambda_i>\prod_{i=0}^n\gamma_i$. If $R>0$

then (18) yields

$$\begin{aligned}\lambda_0(1-e_1) &> \gamma_0 e_1,\\ \lambda_i e_i(1-e_{i+1}) &> \gamma_i e_{i+1}(1-e_i), \quad i=1,\dots,n-1,\\ \lambda_n e_n &> \gamma_n(1-e_n).\end{aligned} \tag{A.11}$$

Multiplying all these inequalities, and using the fact that $e \in \mathrm{Int}(C^n)$ yields

$$\prod_{i=0}^{n} \lambda_i > \prod_{i=0}^{n} \gamma_i. \tag{A.12}$$

To prove the converse implication, assume that (A.12) holds. Multiplying both sides of this inequality by the strictly positive term $\prod_{j=1}^{n} e_j(1-e_j)$ yields

$$\prod_{i=0}^{n} a_i > \prod_{i=0}^{n} b_i,$$

where $a_0 := \lambda_0(1-e_1)$, $a_i := \lambda_i e_i(1-e_{i+1})$, $i=1,\dots,n-1$, $a_n = \lambda_n e_n$, $b_0 := \gamma_0 e_1$, $b_i := \gamma_i e_{i+1}(1-e_i)$, $i=1,\dots,n-1$, and $b_n = \gamma_n(1-e_n)$. This means that $a_\ell > b_\ell$ for some index $\ell \in \{0,\dots,n\}$. Since $R = a_\ell - b_\ell$ (see (18)), it follows that $R > 0$. Summarizing, we showed that $R > 0$ if and only if $\prod_{i=0}^{n} \lambda_i > \prod_{i=0}^{n} \gamma_i$. The proof that $R < 0$ if and only if $\prod_{i=0}^{n} \lambda_i < \prod_{i=0}^{n} \gamma_i$ is similar. This implies that $R = 0$ if and only if $\prod_{i=0}^{n} \lambda_i = \prod_{i=0}^{n} \gamma_i$. This completes the proof of (19).

To prove (20), note that (18) yields

$$\begin{aligned}e_n &= \frac{R+\gamma_n}{\lambda_n+\gamma_n},\\ e_i &= \frac{R+\gamma_i e_{i+1}}{\lambda_i(1-e_{i+1})+\gamma_i e_{i+1}}, \quad i=n-1,\dots,1,\\ e_1 &= \frac{\lambda_0-R}{\lambda_0+\gamma_0}.\end{aligned} \tag{A.13}$$

Substituting $R = 0$ completes the proof of Prop. 5. □

Proof of Theorem 1. The Jacobian of the PMFALK is $J(t, x(t)) = L(t, x(t)) + P(t)$, with L given in (A.3), and P in (A.4) (but now with time-varying rates). Pick an initial time $t_0 \geq 0$, and $\tau_0 > 0$. The stated conditions guarantee the existence of $\zeta \in (0, 1/2)$ such that $x(t, t_0, a) \in C^n_\zeta$ for all $t \geq t_0 + \tau$ and all $a \in C^n$. Also, [4, Thm. 4] implies that there exists a diagonally-scaled L_1 norm such that the PMFALK is contractive on C^n_ζ w.r.t. this norm. Now entrainment follows from known results on contractive systems with a periodic excitation (see, e.g. [7]). □

Proof of Proposition 6. Since the Jacobian $J(x)$ of the MFALK is Metzler (i.e. every off-diagonal entry is non-negative) for any $x \in C^n$, the MFALK is a cooperative system [9], and this yields (24).

When $\lambda_i + \gamma_i > 0$, $i = 1,\dots,n-1$, the matrix $L(x)$ and, therefore, $J(x)$, is irreducible for every $x \in \mathrm{Int}(C^n)$, and combining this with Prop. 1 implies (25) (see, e.g., [9, Ch. 4]). □

Proof of Proposition 7. First, using Remark 1 and the argument used in the proof of [6, Prop. 4] shows that all the derivatives in the statement of of Prop. 7 exist.

Given a MFALK, pick $j \in \{1,\dots,n\}$ and consider the new MFALK obtained by changing α_j to $\tilde{\alpha}_j$, with $\tilde{\alpha}_j > \alpha_j$, and all other rates unchanged. Let $\tilde{e}$, $\tilde{R}$ denote the steady-state density and production rate in the modified MFALK, respectively. Seeking a contradiction, assume that

$$\tilde{e}_n \geq e_n. \tag{A.14}$$

Then (14) implies that

$$\tilde{R} \geq R, \tag{A.15}$$

and if $j = n$ then $\tilde{R} > R$. By (15) with $i = n-1$, $R = \lambda_{n-1}e_{n-1}(1-e_n) - \gamma_{n-1}e_n(1-e_{n-1})$ and $\tilde{R} = \lambda_{n-1}\tilde{e}_{n-1}(1-\tilde{e}_n) - \gamma_{n-1}\tilde{e}_n(1-\tilde{e}_{n-1})$, and combining this with (A.14) and (A.15) yields

$$\tilde{e}_{n-1} \geq e_{n-1}. \tag{A.16}$$

Now using (15) with $i = n-2$ yields $\tilde{e}_{n-2} \geq e_{n-2}$, and $\tilde{e}_{n-2} > e_{n-2}$ if $j = n-1$. Proceeding in this way shows that

$$\tilde{e}_k \geq e_k, \quad k = n, n-1, \dots, j, \tag{A.17}$$

$$\tilde{e}_k > e_k, \quad k = j-1, j-2, \dots, 1. \tag{A.18}$$

Combining this with (15) with $i = 0$ yields $\tilde{R} < R$. This contradicts (A.15), so

$$\tilde{e}_n > e_n. \tag{A.19}$$

Proceeding as above yields $\tilde{e}_i > e_i$ for all i, so $\frac{\partial e_i}{\partial \alpha_j} < 0$ for all i, j. The proofs of all the other equations in Prop. 7 are very similar and therefore omitted. $\square$

References

[1] Z. Aminzare and E. D. Sontag, "Contraction methods for nonlinear systems: A brief introduction and some open problems," in *Proc. 53rd IEEE Conf. on Decision and Control*, Los Angeles, CA, 2014, pp. 3835–3847.

[2] R. A. Blythe and M. R. Evans, "Nonequilibrium steady states of matrix-product form: a solver's guide," *J. Phys. A: Math. Gen.*, vol. 40, no. 46, pp. R333–R441, 2007.

[3] G. Lakatos, T. Chou, and A. Kolomeisky, "Steady-state properties of a totally asymmetric exclusion process with periodic structure," *Phys. Rev. E*, vol. 71, p. 011103, 2005.

[4] M. Margaliot, E. D. Sontag, and T. Tuller, "Entrainment to periodic initiation and transition rates in a computational model for gene translation," *PLoS ONE*, vol. 9, no. 5, p. e96039, 2014.

[5] ——, "Contraction after small transients," *Automatica*, vol. 67, pp. 178–184, 2016.

[6] A. Raveh, M. Margaliot, E. D. Sontag, and T. Tuller, "A model for competition for ribosomes in the cell," *J. Royal Society Interface*, vol. 116, no. 20151062, 2016.

[7] G. Russo, M. di Bernardo, and E. D. Sontag, "Global entrainment of transcriptional systems to periodic inputs," *PLOS Computational Biology*, vol. 6, p. e1000739, 2010.

[8] N. J. A. Sloane and S. Plouffe, *The Encyclopedia of Integer Sequences.* Academic Press, 1995.

[9] H. L. Smith, *Monotone Dynamical Systems: An Introduction to the Theory of Competitive and Cooperative Systems*, ser. Mathematical Surveys and Monographs. Providence, RI: Amer. Math. Soc., 1995, vol. 41.

[10] M. Vidyasagar, *Nonlinear Systems Analysis.* Englewood Cliffs, NJ: Prentice Hall, 1978.
